# Supplementary material for: Streamlined single-molecule RNA-FISH of core clock mRNAs in clock neurons in whole mount Drosophila brains
Source: Front Physiol. 2022 Nov 9;13:1051544. doi: 10.3389/fphys.2022.1051544 (PMC9682093; doi:10.3389/fphys.2022.1051544)
Supplement: Supplementary file 2 [file DataSheet1.pdf]

Supplementary Figure 1

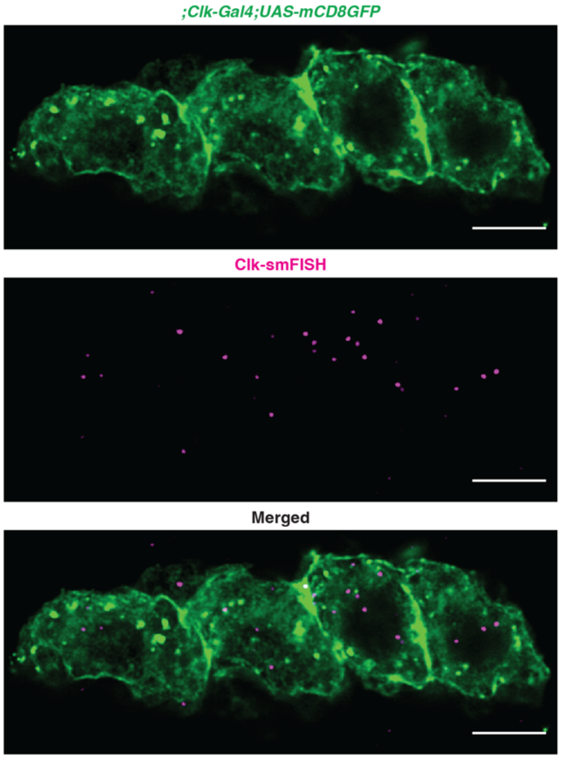

Supplementary Figure S1. Zoomed-out representative image of *Clock* smFISH spots in all lLNvs.

Here we show a representative Z-slice of all four lLNvs observed in a hemi-brain at ZT4. Scale bars, 5 µm.

Supplementary Figure 2

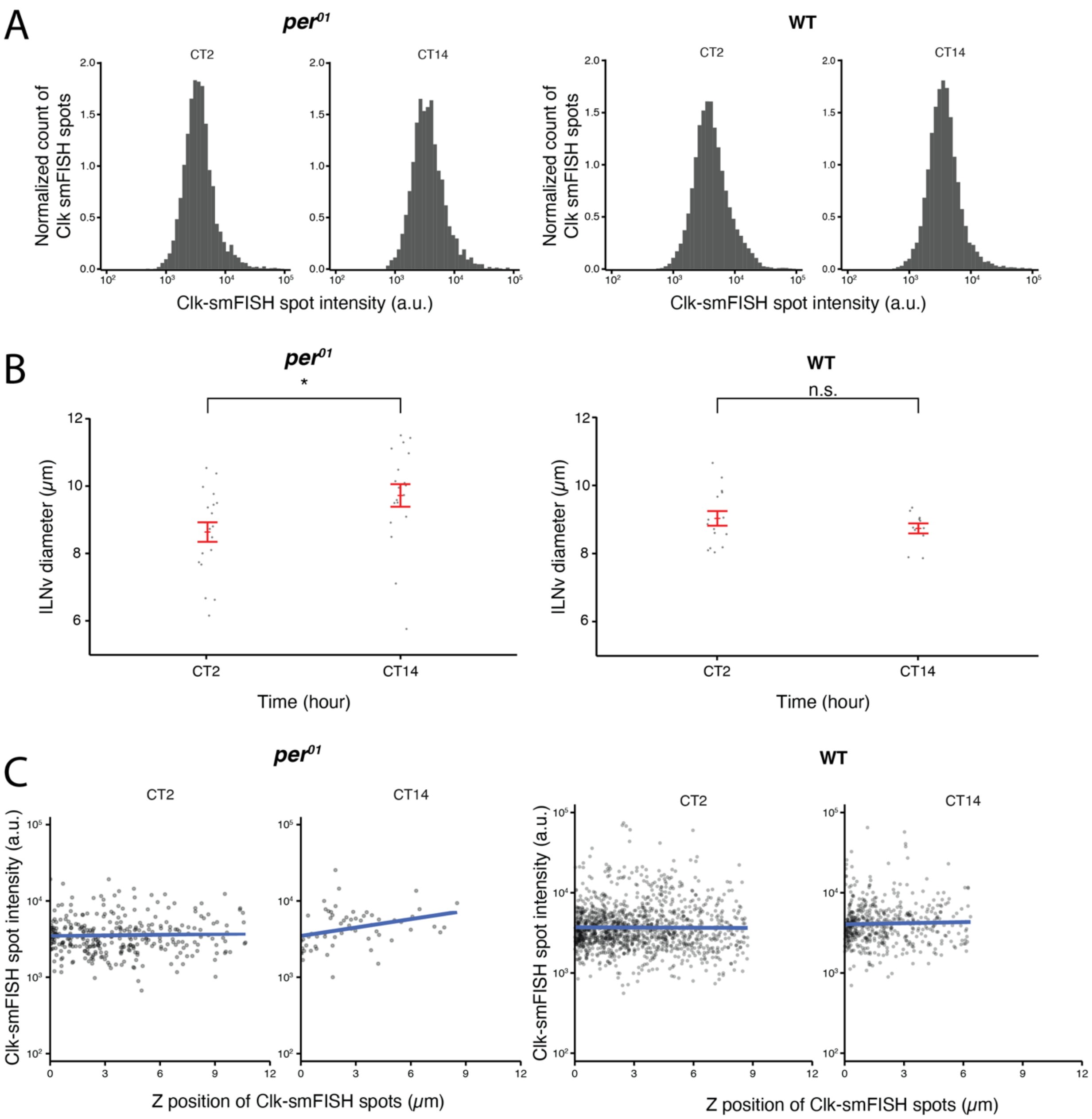

Supplementary Figure S2. Quality control plots for *Clock* smFISH spots in lLNvs from *per*^{01} null mutant flies.

A) Histogram plots of *Clock* smFISH spot intensities in lLNvs from *per*^{01} (left) and wildtype (right) flies at different CT's over the circadian cycle. **B**) Equivalent diameters of lLNvs segmentation masks from *per*^{01} (left) and wildtype (right) flies at different CT's. **C**) *Clock* smFISH spot intensity distribution across all Z-slices of representative lLNvs from *per*^{01} (left) and wildtype (right) flies at different CT's. Statistical test used is unpaired, two-tailed Student's *t*-test assuming unequal variance. *$P < 0.05$, n.s.-not significant. Individual data points, mean, and s.e.m. (standard error of mean) are shown.

Supplementary Figure 3

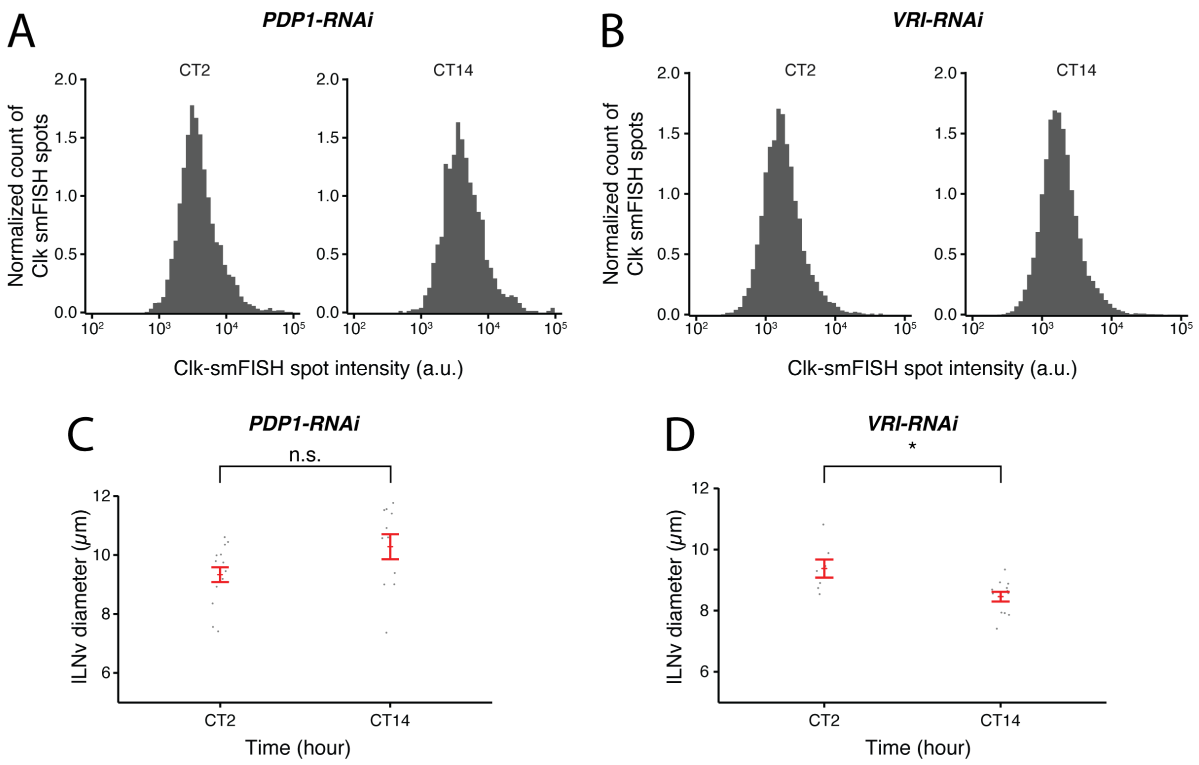

Supplementary Figure S3. Quality control plots for *Clock* smFISH spots in lLNvs from *Clk>Pdp1-RNAi* and *Clk>vri-RNAi* mutants.

A, B) Histogram plots of *Clock* smFISH spot intensities in lLNvs at different CT's over the circadian cycle in PDP-RNAi (A) and VRI-RNAi (B) conditions. **C, D**) Equivalent diameters of lLNvs segmentation masks at different CT's in PDP-RNAi (C) and VRI-RNAi (D) flies. Statistical test used is unpaired, two-tailed Student's *t*-test assuming unequal variance. *$P < 0.05$, n.s.-not significant. Individual data points, mean, and s.e.m. (standard error of mean) are shown.

Supplementary Video 1. Representative segmentation mask of lLNv neurons. A Z-stack video showing a group of 4 lLNv clock neurons marked with CD8-GFP (left panel) and representative segmentation masks from our CNN model (right panel). Scale bar: 4 µm.

Supplementary Video 2. Visualization of detected *Clock* smFISH spots in lLNvs. A Z-stack video showing *Clock* mRNA probe signals in magenta (Quasar 670 fluorophore) and clock neurons in green (CD8-GFP) in the left panel. Right panel shows visualization of detected *Clock* smFISH spots in lLNvs using AIRLOCALIZE algorithm. Scale bar: 4 µm.
